# Metabolomics and Transcriptomics Analysis on Metabolic Characteristics of Oral Lichen Planus

**DOI:** 10.3389/fonc.2021.769163

**Published:** 2021-10-19

**Authors:** Ming-zhe Xin, Ying-ying Shi, Chun-shen Li, Li-hua Zuo, Na Li, Li-wei Liu, He-xin Ma, Qiu-zheng Du, Peng Xue, Zhi Sun, Hong-yu Zhao

**Affiliations:** ^1^ Department of Oral Emergency, The First Affiliated Hospital of Zhengzhou University· Stomatological Hospital of Henan Province, Zhengzhou, China; ^2^ School and Hospital of Stomatology of Zhengzhou University, Zhengzhou, China; ^3^ Department of Pharmacy, The First Affiliated Hospital of Zhengzhou University, Zhengzhou, China; ^4^ Henan Engineering Research Center of Clinical Mass Spectrometry for Precision Medicine, Zhengzhou, China; ^5^ Department of Prosthodontics, The First Affiliated Hospital of Zhengzhou University· Stomatological Hospital of Henan Province, Zhengzhou, China; ^6^ Health Management Center, The First Affiliated Hospital of Zhengzhou University· Stomatological Hospital of Henan Province, Zhengzhou, China

**Keywords:** oral lichen planus, biomarkers, metabolomics, transcriptomics, pathogenesis

## Abstract

**Objective:**

To explore metabolic biomarkers related to erosive and reticulated oral lichen planus (OLP) by non-targeted metabolomics methods and correlate metabolites with gene expression, and to investigate the pathological network pathways of OLP from the perspective of metabolism.

**Methods:**

A total of 153 individuals were enrolled in this study, including 50 patients with erosive oral lichen planus (EOLP), 51 patients with reticulated oral lichen planus (ROLP), and 52 healthy controls (HC). The ultra-high-performance liquid chromatography quadrupole-Orbitrap high-resolution accurate mass spectrometry (UHPLC/Q-Orbitrap HRMS) was used to analyze the metabolites of 40 EOLP, 40 ROLP, and 40 HC samples, and the differential metabolic biomarkers were screened and identified. The regulatory genes were further screened through the shared metabolites between EOLP and ROLP, and cross-correlated with the OLP-related differential genes in the network database. A “gene-metabolite” network was constructed after finding the key differential genes. Finally, the diagnostic efficiency of the biomarkers was verified in the validation set and a diagnostic model was constructed.

**Result:**

Compared with HC group, a total of 19 and 25 differential metabolites were identified in the EOLP group and the ROLP group, respectively. A total of 14 different metabolites were identified between EOLP and ROLP. Two diagnostic models were constructed based on these differential metabolites. There are 14 differential metabolites shared by EOLP and ROLP. The transcriptomics data showed 756 differentially expressed genes, and the final crossover network showed that 19 differential genes were associated with 12 metabolites. Enrichment analysis showed that alanine, aspartate and glutamate metabolism were closely associated with the pathogenesis of OLP.

**Conclusion:**

The metabolic change of different types of OLP were clarified. The potential gene perturbation of OLP was provided. This study provided a strong support for further exploration of the pathogenic mechanism of OLP.

## Introduction

Oral lichen planus (OLP) is one of the most common autoimmune inflammatory diseases (1). The latest research showed that OLP affects 1-2% of the world’s population (2). The clinical manifestations of OLP are diverse (3). The most common type of OLP was the reticulated type (ROLP), followed by the erosion type (EOLP). OLP is a potential malignant disease that can transform into oral squamous cell carcinoma (OSCC) (4, 5). Compared with other types of OLP, erosive OLP is more prone to malignant changes, with a malignant transformation rate of 1.4% (4, 5). Therefore, the early detection and treatment of OLP have great significance in the prevention of oral cancer development. At present, there is a lack of early, minimally invasive and objective screening method for OLP in clinical practice. The treatment of different types of OLP often uses the same strategy, and to a certain extent, the curative effect is poor. In recent years, accumulating evidence indicates the metabolic homeostatic changes in patients with OLP. By understanding the metabolism of different types of OLP, it will help us recognize different types of metabolic targets and open up new avenues for diagnosis and treatment of OLP.

Metabolomics methods have been widely used to characterize the metabolic change and identify the abnormal metabolism in patients with OLP. Yang et al. (6–8) identified multiple metabolic pathways affected by metabolite disorders in serum, urine and tissue samples of reticulated OLP, and constructed a reticulated OLP pathological network based on their findings. Cruz et al. (9)performed metabolomics analysis on erosive and reticulated OLP tissue samples, and found the capability of this technology to distinguish OLP tissue typing. Wang et al. (10) used large sample data to identify the serum metabolites of OLP, and established an OLP biomarker diagnostic model composed of glutamic acid, LysoPE(18:0) and taurine. However, there is no report on the use of liquid chromatography-mass spectrometry technology to comprehensively analyze the difference and connection of serum metabolites between EOLP, ROLP and healthy controls, and there is no correlation between OLP metabolism information and genetic data. The internal gene metabolism network pathway of OLP is not yet clear.

**Figure 1:**
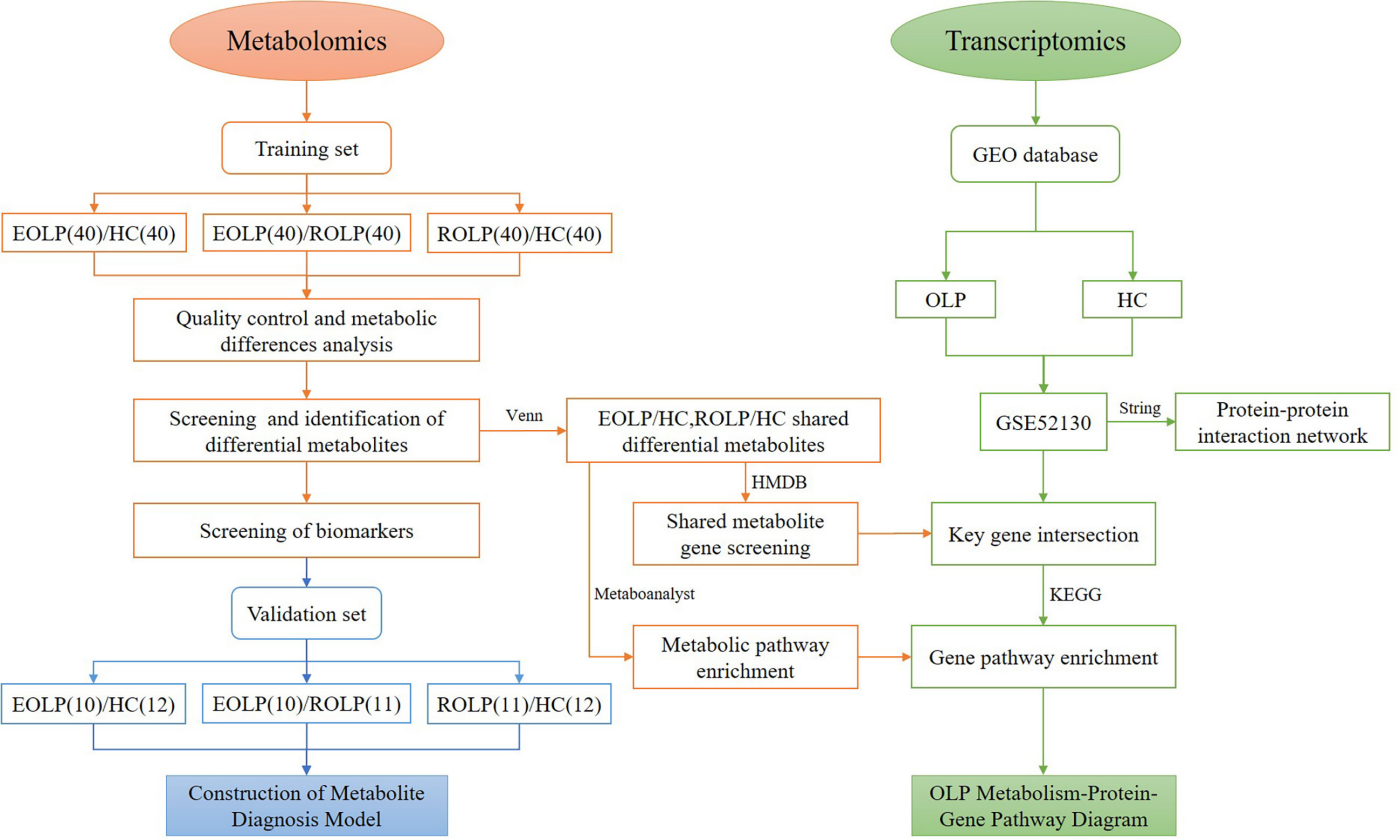
Research flow chart.

In this study, we select patients with EOLP and ROLP as the research subjects. We conduct an in-depth analysis of the metabolic characteristics of their serum samples using the UHPLC/Q-Orbitrap HRMS technology. We identify metabolic biomarkers that are highly correlated with EOLP and ROLP, and clarify the metabolic characteristics of different types of OLP. The common metabolites of EOLP and ROLP were further screened. The transcriptomics data was used to find out common metabolite-related genes and disease related genes. The key genes were used to construct an OLP “metabolite-gene” network to reveal the metabolic characteristics of OLP genes (**Figure 1**). This study will provide a basis for further exploration of the molecular mechanism of oral lichen planus.

## Materials and Methods

### Experimental Materials

#### Experimental Instrument

UHPLC/Q-Orbitrap system: Thermo Fisher Scientific’s Ultra 3000 UHPLC (USA), Thermo Fisher Scientific’s Q Exactive high-resolution mass spectrometer (USA). BX7200HP desktop ultrasonic cleaner from Shanghai Xinmiao Medical Equipment Manufacturing Co., Ltd.; AL104 balance (with an accuracy of 0.0001) from Mettler Toledo Shanghai Co., Ltd., Switzerland; VORTEX-GENIE 2 vortex oscillator from SITM (USA); Hitachi’s CF16RN Centrifuge (Japan); Heraeus Fresco 17 high-speed refrigerated centrifuge from Thermo Fisher Scientific (USA); Ultrapure water meter from MilliPore (USA).

#### Experimental Reagents

Chromatographic grade acetonitrile and methanol were provided by Fisher Company of the United States; Chromatographic grade formic acid was provided by Shanghai Aladdin Biotechnology Co., Ltd.; L-2-chlorophenylalanine was from China Bailingwei Technology Co., Ltd.; Ketoprofen was from Sigma Company of the United States; All solutions were used after being filtered in a 0.22 µm pore filter.

### Experimental Method

#### Participants

A total of 50 EOLP patients and 51 ROLP patients were selected from their first visit to the Department of Stomatology of the First Affiliated Hospital of Zhengzhou University. Patients were diagnosed by clinical and pathological findings. Another 52 healthy individuals (Healthy control, HC) with matching gender and age were selected from the physical examination center of the First Affiliated Hospital of Zhengzhou University. A total of 153 subjects were randomly classified into the training set(n=120) and the validation set(n=33). All patients did not receive any other treatment, such as drug therapy, radiotherapy, chemotherapy, et al. before admission. All subjects had no other oral mucosal diseases, and no basic systemic diseases, such as diabetes, hypertension, and cardiovascular and cerebrovascular diseases. This study was reviewed and approved by the Ethics Committee of the First Affiliated Hospital of Zhengzhou University (serial number: 2019-KY-26/2021-KY-0444), and all subjects signed their informed consents.

#### Sample Collection and Processing

The blood samples were collected from all subjects in the morning after an overnight fasting. The blood samples were collected into a vacuum blood collection tube containing a coagulant and immediately placed in a thermostat with ice cubes. After transfer to the laboratory, blood samples were kept for 30 minutes and centrifuged for 10 minutes at 4°C and 3000 rpm. The supernatant was collected into a new eppendorf tube for aliquoting, and stored in a refrigerator at -80 °C for later use.

#### Sample Pretreatment

Serum samples were thawed on ice. 100 µL of serum was precisely aspirated and placed in a 1.5 mL centrifuge tube, and 300 µL of methanol solution containing internal standard (0.05 μg/mL L-2-chlorophenylalanine and 0.5 μg/mL ketoprofen) was added. Samples were vortexed vigorously for 1 min, and centrifuge at 4°C and 13000 rpm for 10 min. The supernatant was pipetting into the sample injection vial for mass spectrometry analysis. 10 µL samples were separately drawn into 1.5 mL centrifuge tubes as quality control (QC) samples. QC samples were inserted in the process of metabolomics data collection for all samples to ensure the reliability of the experimental results. Before sample analysis, 6 QC samples were first detected, and the stability of the instrument was evaluated by monitoring the pressure changes before and after each injection and the deviation of the retention time of the main peaks in the total ion current graph. After the instrument was stable, the analysis was started. After every 10 samples tested, one QC sample was interspersed. In order to avoid cross-contamination, a blank sample containing only solvent was inserted after each QC sample.

#### System Conditions

The UHPLC system was used to analyze the metabolites in the serum samples. Under the condition of 40°C, 5 µL of liquid was drawn from each sample and injected into the ACQUITY UHPLCBEH C_18_ column (100×2.1 mm, 1.7 µm) for gradient elution. Acetonitrile +0.1% formic acid aqueous solution (A+B) was used as the mobile phase, the flow rate was as follows: 0.2 mL/min: 0~0.5 min, 5%A; 0.5~1.0 min, 5%~60% A; 1.0~7.0 min, 60%~80% A; 7.0~9.0 min, 80~100% A; 9.0~11.0 min, 100%A; 11.0~11.2 min, 100% A-5%A; 11.2~13.0 min, 5%A. The electrospray ionization source (ESI) was used to connect the high-resolution mass spectrometer in series to the UHPLC system. The temperature and flow of the auxiliary gas are 300°C, 10μL/min; the ion source temperature is 350°C; the capillary temperature is 320°C. The scanning range is 80.00~1200.00 m/z. In the Full Mass/ddms 2 scan mode, the detection is performed in the positive and the negative ion mode with a secondary mass spectrum resolution of 17500. The gradient collision energy is 20 eV, 40 eV and 60 eV. The spray voltage and sheath gas flow rate are 3.50 kV and 40 μL/min in the positive ion mode, and 2.80 kV and 38 μL/min in the negative ion mode. The order of injection of all samples is carried out randomly.

#### Identification of Potential Genes Related to Oral Lichen Planus

The oral lichen planus transcriptome data was obtained from the public data platform GEO database (https://www.ncbi.nlm.nih.gov/geo/). 18 samples from the GSE52130 data set were selected, including 10 normal control samples and 8 OLP patient samples. According to the conditions of P<0.05, |Log2(fold change (FC))|>0.6, the differentially expressed genes (DEGs) between OLP and healthy control epithelial tissues were screened. The STRING database (https://www.string-db.org/) was used to make a protein-protein interaction (PPI) network, and the Cytoscape 3.8.2 software was used to visualize the network graphics. The crossed genes were tested using a Venn diagram.

#### Statistical Analysis

The mass spectrometry data is analyzed by the Thermo Xcalibur™ software. Compound Discoverer software 3.0 was used for peak calibration, peak matching and peak alignment information extraction. The SIMCA 14.1 (Umetrics, Sweden) software was used to perform multivariate statistical analysis on all samples, including principal component analysis (PCA), orthogonal projection to latent structure discriminant analysis (OPLS-DA). Simultaneously, 200 permutation tests were performed to evaluate the overfitting of the analysis. The result of the OPLS-DA is the estimated value of the variable importance in the projection (VIP). The metabolites were screened according to the conditions of P<0.05, FC>2 and VIP>1, and the screened metabolites were compared with the Human Metabolome Database (HMDB) (https://hmdb.ca/), the mzCloud (https://www.mzcloud.org/), and the PubChem database (https://pubchem.ncbi.nlm.nih.gov/). The laboratory’s self-built database mzVault was used to compare various information, including molecular formula, molecular weight, retention time, secondary fragment structure information. The MetaboAnalyst (https://www.metaboanalyst.ca/) was used to produce metabolite volcano maps, heat maps, receiver operating characteristic curve (ROC), and calculate the area under the curve (AUC). SPSS 25.0 (IBM, USA) software was used to calculate the visual correlation coefficient, and Cytoscape software 3.8.2 was used for visualization. The Kyoto Encyclopedia of Genes and Genomes (KEGG) (http://www.genome.jp/kegg/) was used to enrich the metabolic pathways. Transcriptomics data is integrated through MetaboAnalyst.

## Results

### Analysis of Population Baseline Characteristics

A total of 120 participants were selected in in the training set, including 40 EOLPs, 40 ROLPs, and 40 healthy control individuals. The validation set selected a total of participants, including 10 EOLPs, 11 ROLPs, and 12 healthy control individuals. There was no significant difference in the baseline characteristics between the diseased group and the healthy control group (P>0.05) (**Table 1**).

**Table 1:** The baseline characteristics of participants.

	Training set			Validation set			P value	
	EOLP (n = 40)	ROLP (n = 40)	HC (n = 40)	EOLP (n = 10)	ROLP (n = 11)	HC (n = 12)	Training set	Validation set
male/female	15/25	17/23	13/27	3/7	3/8	4/8	0.65	0.95
Age(years)	51.43 ± 12.54	52.47 ± 14.32	50.43 ± 9.76	52.92 ± 7.67	51.46 ± 11.30	51.08 ± 7.76	0.59	0.12
BMI (kg/m^2^)	24.13 ± 3.10	24.31 ± 4.94	23.34 ± 2.85	24.88 ± 4.03	23.25 ± 3.81	22.97 ± 2.69	0.47	0.91
Smoking history	8	10	7	1	1	1	0.70	0.99
Drinking history	7	15	14	2	2	2	0.10	0.98
Lack of exercise	18	23	18	5	5	5	0.43	0.99
Like spicy food	14	18	17	6	5	5	0.63	0.67
Like hot food	19	22	13	5	5	4	0.12	0.71
Lack of sleep	8	10	3	3	4	0	0.11	0.07

BMI, body mass index; Data are presented as the mean ± SD.

### Quality Control and Metabolic Difference

**Figure 2:**
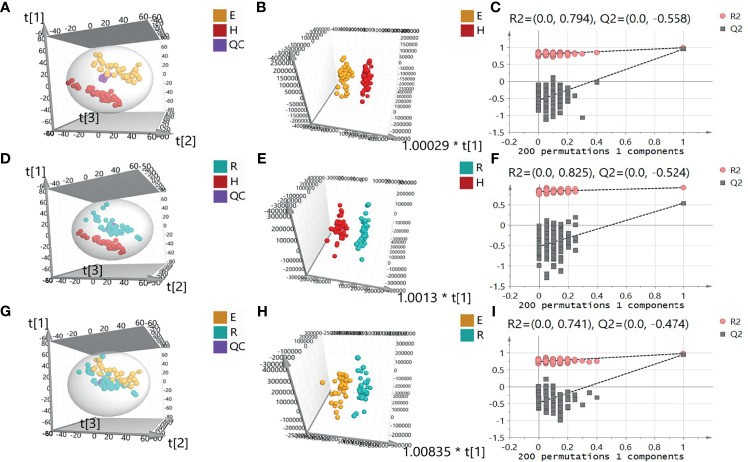
Principal component analysis (PCA) diagram of serum samples of EOLP group and HC group **(A)**, ROLP group and HC group **(D)**, EOLP group and ROLP group **(G)** in the negative ion mode, OPLS-DA score diagram **(B, E, H)** and 200 permutation test **(C, F, I)** of EOLP group and HC group, ROLP group and HC group, EOLP group and ROLP group in the negative ion mode. E: EOLP; R: ROLP; H: HC.

A total of 5450 ion peaks were detected in the serum samples in the negative ion mode. Principal component analysis (PCA) showed that the QC samples are tightly gathered in the negative ion mode, which indicates that the instrument is working normal and the detection data is stable and reliable. The results of the EOLP group, ROLP group, and HC group were well separated (**Figures 2A, B**), indicating that there are significant differences among the three groups. The results of OPLS-DA showed the metabolic differences between two groups (**Figures 2C, D**). The results of 200 permutation tests of the OPLS-DA showed that both the R2 and Q2 values were good, suggesting that the OPLS-DA did not have over-fitting phenomenon (**Figures 2E, F**), and the OPLS-DA results have high credibility. A similar separation result was also shown in the positive ion mode (**Supplementary Figure 1**).

### Screening and Identification of Differential Metabolites

#### Screening of Differential Metabolites

**Figure 3:**
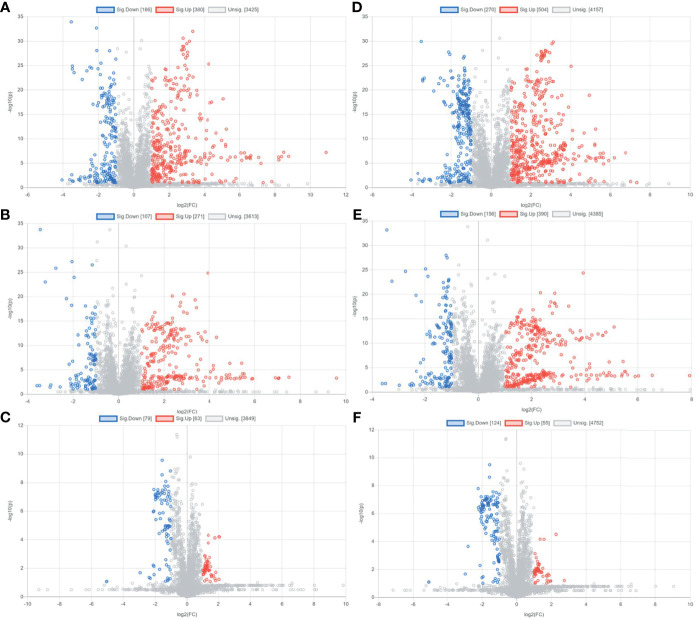
Metabolite volcano analysis diagram of EOLP group and HC group **(A)**, ROLP group and HC group **(B)**, EOLP group and ROLP group **(C)** in the negative ion mode; and EOLP group and HC group **(D)**, ROLP group and HC group **(E)**, EOLP group and ROLP group **(F)** in the positive ion mode.

Preliminary screening of differential metabolites by P value (P <0.05) and FC value (FC >2.0) was performed and a volcano plot was drawn (**Figure 3**). The results showed that among the effective metabolites screened, 186 were down-regulated and 380 were up-regulated in the EOLP group compared with the HC group in the negative ion mode (**Figure 3A**); 107 were down-regulated and 271 were up-regulated in the ROLP group compared with the HC group (**Figure 3B**); 79 were down-regulated and 63 were up-regulated in the EOLP group compared with the ROLP group (**Figure 3C**) In the positive ion mode, there were 270 down-regulated metabolites and 504 up-regulated metabolites in the EOLP group, (**Figure 3D**); 156 down-regulated metabolites and 390 up-regulated metabolites in the ROLP group compared with the HC group (**Figure 3E**); 124 down-regulated metabolites and 55 up-regulated metabolites in the EOLP group compared with the ROLP group (**Figure 3F**). The above-mentioned differential metabolites were further screened based on the VIP>1 condition.

#### Identification of Differential Metabolites

**Figure 4:**
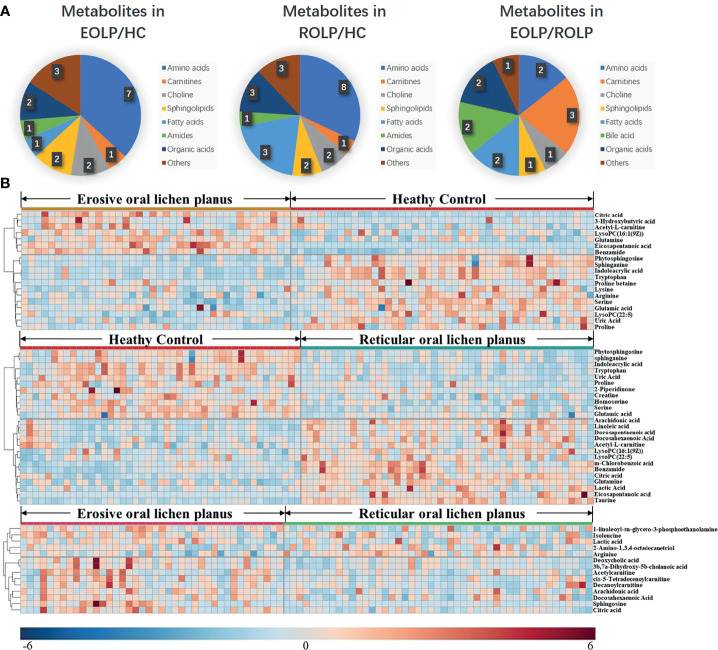
The pie chart shows the distribution of different metabolites **(A)**. The heat map analysis of endogenous metabolites in the EOLP group and healthy group, ROLP and HC group, EOLP group and ROLP group **(B)**.

The molecular formula, molecular weight and secondary structure of the selected candidate metabolites were compared with the data in the database. After excluding unqualified metabolites, 19 endogenous compounds were finally identified in the EOLP and HC groups (**Table 2**), and 25 endogenous compounds were finally identified in the ROLP and HC groups (**Table 3**), and 14 endogenous compounds were finally identified in the EOLP and ROLP groups (**Table 4**). The types of metabolites include amino acids, fatty acids, sphingolipids and other small molecule compounds (**Figure 4A**). The heat map analysis of the different metabolites showed that each metabolite was significantly different in the two sets of samples (**Figure 4B**). Spearman correlation analysis was used to further explore the correlation between the differentially expressed metabolites. The results showed that there is a strong correlation between the differential metabolites and the EOLP group and the ROLP group (**Supplementary Figure 2**).

**Table 2:** Differential metabolites between EOLP group and healthy group.

No.	Metabolite name	Ion type	Molecular formula	Theoretical m/z	Experimental m/z	Error (ppm)	RT (min)	VIP	FC	P-value	AUC
1	Eicosapentanoic acid	N	C_20_H_30_O_2_	301.21730	301.21777	1.563	9.64	1.65	17.11	2.05E-12	0.993
2	Citric acid	N	C_6_H_8_O_7_	192.02755	191.01950	0.871	1.27	2.86	2.49	6.97E-15	0.958
3	Serine	P	C_3_H_7_NO_3_	106.04987	106.05020	0.330	1.09	2.49	0.77	1.66E-13	0.902
4	Sphinganine	P	C_18_H_39_NO_2_	302.30536	302.30536	0.004	7.34	2.95	0.71	1.71E-08	0.894
5	Indoleacrylic acid	P	C_11_H_9_NO_2_	188.07061	188.07069	0.085	3.38	12.72	0.67	8.92E-13	0.891
6	Tryptophan	P	C_11_H_12_N_2_O_2_	205.09715	205.09729	0.136	3.38	12.73	0.68	1.20E-12	0.888
7	Phytosphingosine	P	C_18_H_39_NO_3_	318.30027	318.30020	-0.071	6.72	4.50	0.64	2.48E-09	0.886
8	Benzamide	P	C_7_H_7_NO	122.06004	122.06013	0.090	3.77	3.08	1.74	8.89E-09	0.874
9	Uric Acid	P	C_5_H_4_N_4_O_3_	169.03562	169.03561	-0.007	1.27	4.25	0.71	4.23E-06	0.783
10	Glutamine	N	C_5_H_10_N_2_O_3_	145.06187	145.06104	0.271	0.92	1.40	1.38	4.74E-06	0.761
11	LysoPC(22:5)	P	C_30_H_52_NO_7_ P	570.35542	570.35614	0.724	8.45	1.05	0.70	6.58E-04	0.740
12	Lysine	P	C_6_H_14_N_2_O_2_	147.11280	147.11278	-0.024	0.83	1.15	0.81	1.05E-03	0.734
13	LysoPC(16:1(9Z))	P	C_24_H_48_NO_7_ P	494.32412	494.32553	1.414	8.06	1.54	1.15	4.19E-02	0.732
14	3-Hydroxybutyric acid	N	C_4_H_8_O_3_	103.04007	103.03893	-0.041	1.70	1.19	2.27	3.41E-03	0.726
15	Glutamic acid	P	C_5_H_9_NO_4_	148.06043	148.06038	-0.054	1.07	1.18	0.87	6.31E-03	0.719
16	Acetyl-L-carnitine	P	C_9_H_17_NO_4_	204.12303	204.12286	-0.175	1.13	4.11	1.76	5.68E-06	0.718
17	Arginine	P	C_6_H_14_N_4_O_2_	175.11895	175.11903	0.078	0.85	2.38	0.79	6.97E-04	0.707
18	Proline betaine	P	C_7_H_13_NO_2_	144.10191	144.10188	-0.025	0.97	2.15	0.51	3.12E-02	0.697
19	Proline	P	C_5_H_9_NO_2_	116.07061	116.07075	0.145	0.96	3.15	0.76	8.10E-04	0.690

**Table 3:** Differential metabolites between ROLP group and healthy group.

No.	Metabolite name	Ion type	Molecular formula	Theoretical m/z	Experimental m/z	Error (ppm)	RT (min)	VIP	FC	P-value	AUC
1	Eicosapentanoic acid	N	C_20_H_30_O_2_	301.21730	301.21771	1.503	9.64	1.93	18.13	1.34E-06	0.976
2	Taurine	N	C_2_H_7_NO_3_S	124.00739	124.00643	0.140	0.96	1.90	2.02	5.97E-14	0.934
3	Serine	P	C_3_H_7_NO_3_	106.04987	106.05020	0.330	1.09	2.98	0.78	7.74E-13	0.905
4	Arachidonic acid	N	C_20_H_32_O_2_	303.23295	303.23334	1.483	10.08	5.25	2.36	4.55E-10	0.884
5	Citric acid	N	C_6_H_8_O_7_	191.01972	191.01956	0.931	1.27	2.45	1.77	1.14E-13	0.880
6	Benzamide	P	C_7_H_7_NO	122.06004	122.06010	0.060	3.77	4.84	1.71	3.46E-08	0.871
7	Indoleacrylic acid	P	C_11_H_9_NO_2_	188.07061	188.07072	0.115	3.38	13.79	0.72	1.29E-10	0.855
8	Tryptophan	P	C_11_H_12_N_2_O_2_	205.09715	205.09729	0.136	3.38	13.78	0.73	1.85E-10	0.852
9	Uric Acid	P	C_5_H_4_N_4_O_3_	169.03562	169.03561	-0.007	1.27	5.55	0.67	2.56E-07	0.815
10	Phytosphingosine	P	C_18_H_39_NO_3_	318.30027	318.30026	-0.011	6.72	3.85	0.68	9.81E-08	0.798
11	m-Chlorobenzoic acid	N	C_7_H_5_ClO_2_	154.99053	154.98967	0.236	6.18	1.19	1.30	7.60E-05	0.791
12	Docosapentaenoic acid	N	C_22_H_34_O_2_	329.24860	329.24899	1.483	10.29	1.44	2.48	1.34E-06	0.789
13	Linoleic acid	N	C_18_H_32_O_2_	279.23295	279.23322	1.363	10.22	5.76	1.63	5.85E-07	0.774
14	Homoserine	P	C_4_H_9_NO_3_	120.06552	120.06572	0.200	1.07	1.20	0.77	1.23E-05	0.773
15	Glutamine	N	C_5_H_10_N_2_O_3_	145.06187	145.06105	0.281	0.97	1.81	1.39	4.33E-07	0.749
16	sphinganine	P	C_18_H_39_NO_2_	302.30536	302.30536	0.004	7.34	2.03	0.78	7.83E-05	0.743
17	Glutamic acid	P	C_5_H_9_NO_4_	148.06043	148.06038	-0.054	1.07	1.68	0.85	3.85E-04	0.742
18	Docosahexaenoic Acid	N	C_22_H_32_O_2_	327.23295	327.23364	1.783	9.90	1.85	1.46	7.19E-04	0.713
19	Lactic Acid	N	C_3_H_6_O_3_	89.02441	89.02323	-0.091	1.13	2.08	1.29	3.10E-03	0.696
20	Proline	P	C_5_H_9_NO_2_	116.07061	116.07075	0.145	0.96	3.56	0.79	6.15E-03	0.694
21	LysoPC(16:1(9Z))	P	C_24_H_48_NO_7_ P	494.32412	494.32562	1.504	8.06	2.30	1.18	2.14E-02	0.692
22	Creatine	P	C_4_H_9_N_3_O_2_	132.07683	132.07683	0.077	0.96	2.14	0.88	1.57E-03	0.687
23	2-Piperidinone	P	C_5_H_9_NO	100.07569	100.07612	0.429	2.16	2.33	0.44	7.96E-03	0.664
24	LysoPC(22:5)	P	C_30_H_52_NO_7_ P	570.35542	570.35614	0.724	8.45	2.06	1.19	3.91E-02	0.658
25	Acetyl-L-carnitine	P	C_9_H_17_NO_4_	204.12303	204.12286	-0.175	1.13	2.44	1.36	1.54E-03	0.656

**Table 4:** Differential metabolite identification results between EOLP group and ROLP group.

No.	Metabolite name	Ion type	Molecular formula	Theoretical m/z	Experimental m/z	Error(ppm)	RT(min)	VIP	FC	P-value	AUC
1	Sphingosine	P	C_18_H_37_NO_2_	300.28970	300.29013	0.424	7.63	2.21	2.99	6.88E-05	0.760
2	Deoxycholic acid	N	C_24_H_40_O_4_	391.28538	391.28616	1.874	9.84	1.82	1.52	1.79E-04	0.756
3	3b,7a-Dihydroxy-5b-cholanoic acid	P	C_24_H_40_O_4_	393.29993	393.30002	0.084	9.87	1.65	1.48	4.71E-04	0.750
4	1-linoleoyl-sn-glycero-3-phosphoethanolamine	P	C_23_H_44_NO_7_ P	478.29282	478.29239	0.493	8.30	3.05	1.14	5.05E-02	0.683
5	Citric acid	N	C_6_H_8_O_7_	191.01972	191.01955	0.921	1.27	1.78	1.33	5.50E-04	0.675
6	Arachidonic acid	N	C_20_H_32_O_2_	303.23295	303.23340	1.543	10.08	2.69	1.32	2.97E-03	0.673
7	Isoleucine	P	C_6_H_13_NO_2_	132.10190	132.10194	0.035	1.48	7.85	1.07	5.85E-03	0.672
8	cis-5-Tetradecenoylcarnitine	P	C_21_H_39_NO_4_	370.29518	370.29529	0.105	7.36	1.10	1.50	1.65E-03	0.661
9	Decanoylcarnitine	P	C_17_H_33_NO_4_	316.24823	316.24838	0.145	6.38	1.57	1.29	8.55E-02	0.657
10	Lactic acid	N	C_3_H_6_O_3_	89.02441	89.02323	-0.091	1.27	5.20	1.12	4.29E-02	0.643
11	Docosahexaenoic Acid	N	C_22_H_32_O_2_	327.23295	327.23364	1.783	9.90	1.08	1.22	4.48E-03	0.640
12	2-Amino-1,3,4-octadecanetriol	P	C_18_H_39_NO_3_	318.30027	318.30023	-0.041	6.76	2.25	0.93	1.27E-02	0.619
13	Arginine	P	C_6_H_14_N_4_O_2_	175.11895	175.11903	0.078	0.99	1.93	0.85	1.26E-02	0.603
14	Acetylcarnitine	P	C_9_H_17_NO_4_	204.12303	204.12291	-0.125	1.13	2.62	1.31	7.33E-03	0.586

### Correlation of Important Metabolites in EOLP and ROLP

**Figure 5:**
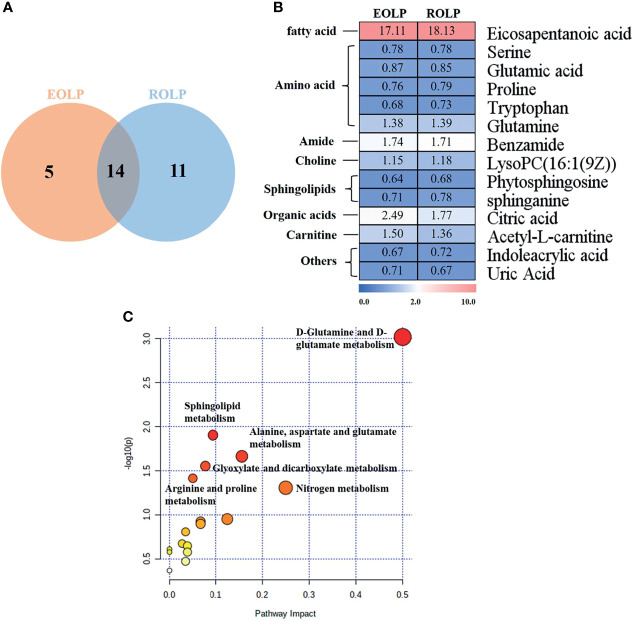
Venn diagram of EOLP and ROLP metabolites overlapped **(A)**, Fold change analysis (red, up-regulated; blue, down-regulated) **(B)**, Metabolism pathway diagram of common metabolites in EOLP and ROLP groups **(C)**.

A total of 14 key metabolites with differential expression in EOLP and ROLP were found, including glutamic acid, tryptophan, indoleacrylic acid and other metabolites (**Figures 5A, B**). The eicosapentanoic acid, citric acid, and benzamide were positively correlated in the EOLP and ROLP groups (**Figure 5B**). In order to explore the connection of different metabolites, Spearman correlation coefficient was used to analyze the correlation of these 14 metabolites (**Supplementary Figure 3**). The metabolic pathways of shared metabolites were identified and six important metabolic pathways were identified, including: D-glutamine and D-glutamate metabolism, sphingolipid metabolism; alanine, aspartate and glutamate metabolism; arginine and proline metabolism; glyoxylate and dicarboxylate metabolism; nitrogen metabolism (**Figure 5C**). It can be seen that the metabolic changes were closely associated with abnormal amino acid metabolism in patients with OLP.

### Construction of a Genetic Biological Network Related to Differential Metabolites

**Figure 6:**
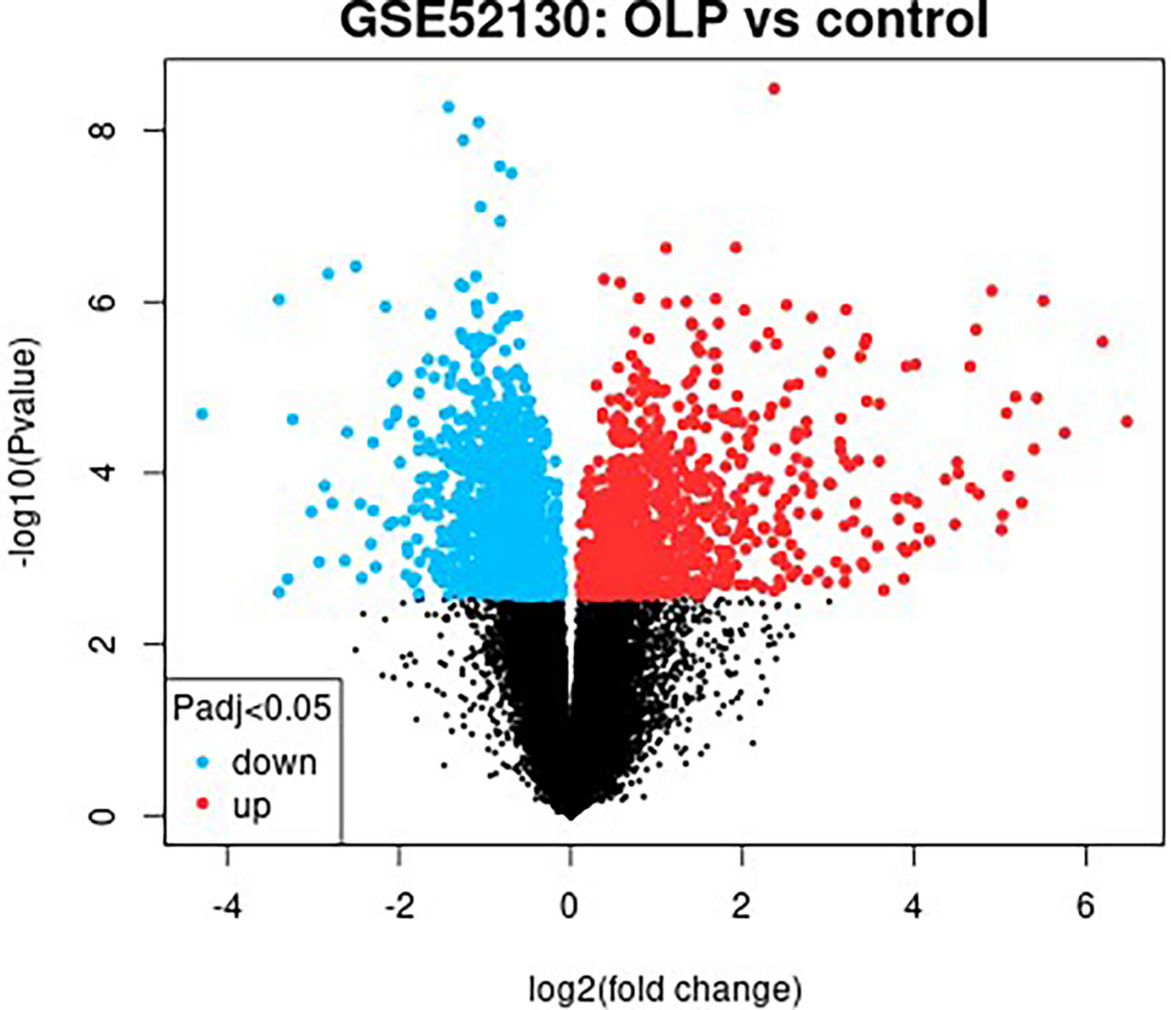
Differential gene screening results of the OLP and HC groups in the GSE52130 data set.

**Figure 7:**
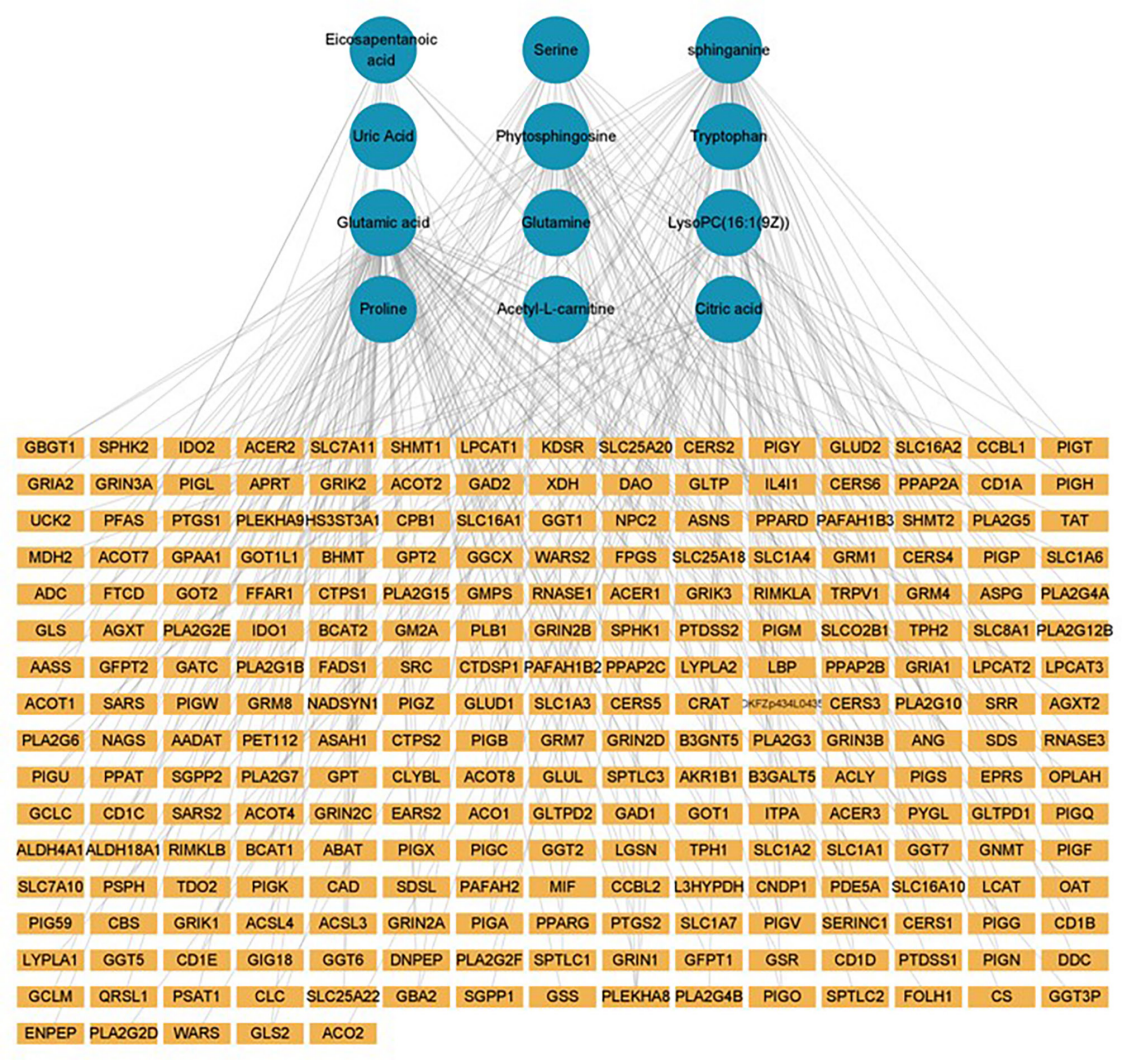
Metabolite-gene network (green circle: metabolite, yellow rectangle: gene).

**Figure 8:**
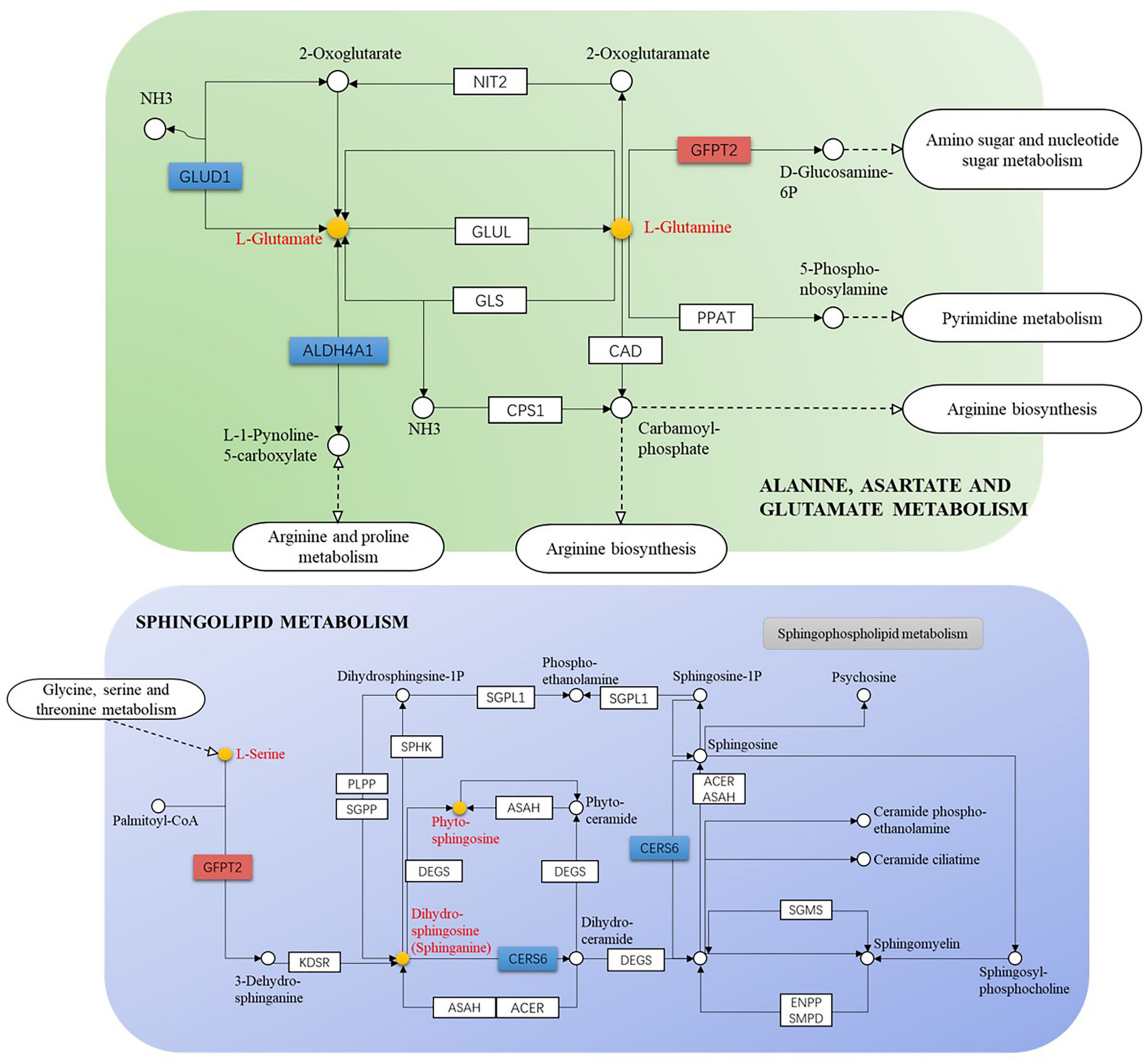
Gene-metabolite connection network diagram (yellow circle: key metabolite, blue rectangle: key protein level decreased, orange rectangle: key protein level increased).

The GSE52130 dataset was selected from the GEO database and successfully screened out 2013 different genes between the OLP and HC groups (adjusted P value<0.05). Among them, 782 genes were up-regulated and 1231 genes were down-regulated (**Figure 6**). The 1227 DEGs with |Log2FC|>0.6 were selected to construct a PPI network using the STRING database (**Supplementary Figure 4**). HMDB and KEGG databases were used and 223 genes were found to be closely related to 14 common differential metabolites. Among them, the relevant gene information of benzamide and indoleacrylic acid was not found. The Cytoscape software was used to construct a “metabolite-gene” network (**Figure 7**). CERS6, SPTLC3, PIGQ and 19 other genes were highlighted by crossing the GSE52130 data set and the metabolite genes network (**Table 5**). The results showed that a total of 3 pathways in OLP received significant interference (**Table 6**). Gene pathway and metabolic pathway jointly participate in the processes of alanine, aspartate and glutamate metabolism and sphingolipid metabolism. The relationship between genes and metabolism was explored by searching the KEGG and HMDB online databases, and the relevant metabolites and key genes were displayed in the network diagram (**Figure 8**).

**Table 5:** The key targets identified by integration analysis of metabolomic and transcriptomic data.

No.	Gene.symbol	Gene.title	Related metabolites
1	CERS6	ceramide synthase 6	Sphinganine
2	SPTLC3	serine palmitoyltransferase long chain base subunit 3	Serine
3	SLC1A4	solute carrier family 1 member 4	Serine
4	PIGQ	phosphatidylinositol glycan anchor biosynthesis class Q	Phytosphingosine
5	PIGS	phosphatidylinositol glycan anchor biosynthesis class S	Phytosphingosine
6	PAFAH1B3	platelet activating factor acetylhydrolase 1b catalytic subunit 3	LysoPC(16:1(9Z))
7	DNPEP	aspartyl aminopeptidase	Glutamic acid
8	ALDH4A1	aldehyde dehydrogenase 4 family member A1	Glutamic acid
9	GFPT2	glutamine-fructose-6-phosphate transaminase 2	Glutamic acid
10	OPLAH	5-oxoprolinase (ATP-hydrolysing)	Glutamic acid
11	GLUD1	glutamate dehydrogenase 1	Glutamic acid
12	BCAT2	branched chain amino acid transaminase 2	Glutamic acid
13	PTGS1	prostaglandin-endoperoxide synthase 1	Eicosapentanoic acid
14	PTGS2	prostaglandin-endoperoxide synthase 2	Eicosapentanoic acid
15	FADS1	fatty acid desaturase 1	Eicosapentanoic acid
16	AKR1B1	aldo-keto reductase family 1 member B	Citric acid
17	CTDSP1	CTD small phosphatase 1	Citric acid
18	CLYBL	citrate lyase beta like	Citric acid
19	CRAT	carnitine O-acetyltransferase	Acetyl-L-carnitine

**Table 6:** KEGG enrichment pathway of genes.

Term	ID	Count	P-Value	FDR	Input
hsa00250	Alanine, aspartate and glutamate metabolism	3	2.20E-03	4.74E-02	ALDH4A1↓, GLUD1↓, GFPT2↑
hsa00600	Sphingolipid metabolism	2	9.16E-02	8.41E-01	CERS6↓, SPTLC3↑
hsa00563	Glycosylphosphatidylinositol (GPI)-anchor biosynthesis	2	4.97E-02	7.13E-01	PIGS↓, PIGQ↓

### Construction of Metabolite Diagnostic Model

**Figure 9:**
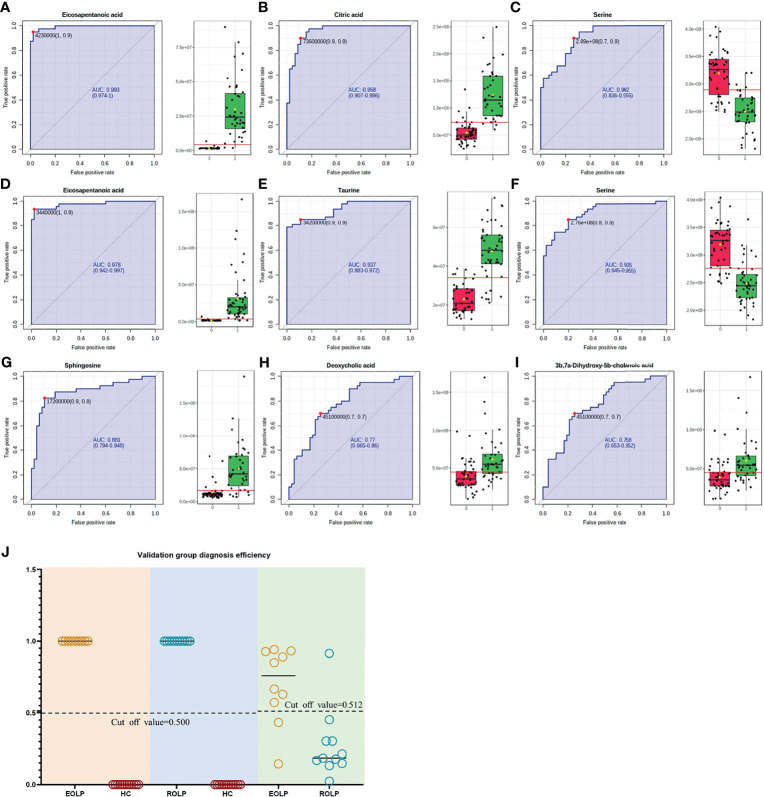
ROC curves of Eicosapentanoic acid **(A)**, Citric acid **(B)**, and Serine **(C)** in the EOLP group and HC group; Eicosapentanoic acid **(D)**, Taurine **(E)**, and Serine **(F)** in the ROLP group and HC group; Sphingosine **(G)**, deoxycholic acid **(H)**, and 3b,7a-Dihydroxy-5b-cholanoic acid **(I)** in the EOLP group and ROLP group. Validation group diagnosis efficiency **(J)**.

The AUC of the EOLP and ROLP metabolites were calculated, and the results were shown in **Tables 2**, **3**, and **4**, respectively. According to the ranking of the AUC value combined with the VIP value, eicosapentanoic acid, citric acid, and serine were finally selected as the biomarkers between EOLP and HC (**Figures 9A–C**). Eicosapentanoic acid, taurine, and serine are the biomarkers between ROLP and HC (**Figures 9D–F**). Sphingosine, deoxycholic acid, and 3b,7a-Dihydroxy-5b-cholanoic acid are the biomarkers between EOLP and ROLP (**Figures 9G–I**). The binary Logistic regression is used to evaluate the diagnostic efficacy of the model in the validation group. According to the predictive sensitivity and specificity of the model, the test variable value with the highest Youden index (EOLP/HC=0.500, ROLP/HC=0.500, EOLP/ROLP=0.512) is selected as the best diagnostic cut-off value, EOLP and HC group. The diagnostic accuracy of ROLP and HC groups reached 100%, and the diagnostic accuracy of EOLP and ROLP reached 92.68% (**Figure 9J**).

## Discussion

The metabolic data of serum samples from EOLP, ROLP, and healthy controls were comprehensively analyzed based on the UHPLC/Q-Orbitrap HRMS technology. The metabolic phenotype changes of the two types of OLP were clarified, and the ideal diagnostic models of EOLP and ROLP were constructed respectively. We used transcriptomics data to identify key genes that share differentially expressed metabolites, and found the key signaling pathways and metabolic pathways involved in OLP. This study not only reveals the regulatory characteristics of different types of OLP metabolic networks, but also explores the key the related pathological processes of OLP, and elucidates the gene metabolism network of OLP.

Among many different metabolites, we found that eicosapentanoic acid (EPA) is the most variable biomarker in EOLP and ROLP. It was known that the pathogenesis of OLP is closely related to changes in immune function and inflammation (11, 12). Chronic inflammation is one of the reasons of tumorigenesis (13, 14). Studies have shown that EPA can improve human immune function and has anti-inflammatory effects (15). This study found that the content of EPA in the diseased group was significantly higher than that in the healthy control group (FC<1), which may be related to the increased inflammation and immune dysfunction of OLP. In addition, EPA can inhibit the production of inflammatory cytokines, such as tumor necrosis factor (TNF-α), and in turn affect its downstream activities, including the inhibition of NF-κB (16). NF-κB plays a key role in the process of cellular inflammation and immune response (17). The mis-regulation of NF-κB can cause autoimmune diseases, chronic inflammation and cancers (18–20). Studies have shown that TNF-α is overexpressed in OLP patients, and the metabolic changes of EPA may be related to the high expression of TNF-α and the activation of the NF-κB signaling pathway. Interestingly, EPA has a greater fold change in the EOLP group, so we speculate that the increase in EPA is likely to indicate the progression of the disease, which requires further research.

In this study, the content of glutamic acid decreased in the OLP group. Glutamic acid is believed to play a significant role in regulating the body’s oxidative stress (21). Oxidative stress accompanied by inflammation will aggravate the onset and progression of OLP (22). Abnormal metabolism of glutamate and hypoxanthine may induce oxidative stress in the body, leading to OLP. Previous studies suggest that most patients with OLP have emotional stress such as insomnia and anxiety, and mental factors can stimulate the occurrence and development of OLP (23). In this study, it was found that more people lacked of sleep in the OLP group. Tryptophan is the precursor of serotonin (5-HT) produced in the periphery and the central nervous system (24, 25). Serotonin has long been considered an important regulator of mood, especially in the pathophysiology of depression (26). A large number of studies have shown that 5-HT can reduce the level of tryptophan by reducing the availability of its precursor (27). In this study, it was found that tryptophan levels decreased to varying degrees in the EOLP and ROLP groups, and the level of decrease in EOLP was slightly higher than that in the ROLP group. We speculate that the level of tryptophan may indirectly affects the emotional status of OLP patients, thereby intervening the progression of the disease.

This study found that sphingolipids in OLP patients were lower than those in the healthy control group, and the sphingolipid metabolism pathway may be involved in the pathogenesis of OLP. Sphingolipids and their metabolites are not only important structural molecules that constitute cell membranes, but also participate in many important signal transduction processes such as the regulation of cell growth, differentiation, senescence and programmed cell death (28, 29). The decrease in the levels of sphinganine and phytosphingosine in OLP patients may be one of the important reasons for inducing cell destruction and apoptosis. Other studies have shown that many sphingolipid regulators (such as sphinganine) are closely related to the occurrence and development of cancers (30). OLP could progress to malignant disease, and the relationship between the reduction of sphingolipids and OLP carcinogenesis remains to be further explored.

In addition, several key regulatory genes were found after integrating the transcriptomics data, which provided evidence for the biosynthetic pathways and regulatory mechanisms of key metabolites. The canonical role of glutamate dehydrogenase 1 (GLUD1) is a consequence of the efficient transference by transaminases of the α-amino group of several amino acids to 2-OG forming glutamic acid (31). Therefore, the decrease in GLUD1 level may be one of the direct reasons for the decrease in glutamic acid content. The mRNA level of GFPT2 has been up-regulated in many cancers such as glioblastoma, lung adenocarcinoma and breast cancer (32, 33). This study found that the GFPT2 level was up-regulated in OLP patients, which may be the regulatory mechanism related to OLP malignant transformation.

This study provides a new perspective for the diagnosis and pathological mechanism of the two types of OLP, but it has some limitations. Although the diagnostic metabolites of EOLP and ROLP have been identified in this study, the sample size of the data is not large enough and comes from the same center. The extensiveness and specificity of diagnostic markers still need a larger sample size for further research. Also, the transcriptomics data in this article are derived from online database, and lack of experimental validation, which may impact final results. However, through the integration of transcriptomics and metabolomics data, this article provides certain information on the relationship between metabolites and DEGs, and comprehensively clarifies the metabolic characteristics of OLP.

In summary, compared with HC group, this study identified 19 and 25 differential metabolites in patients with EOLP and ROLP, respectively. A total of 14 different metabolites were identified between EOLP and ROLP. Two important metabolic pathways directly related to OLP have been found. A model of potential metabolic diagnostic markers of EOLP, ROLP and HC was constructed. These findings clarify the characteristics of OLP gene metabolism, and provide guidance for further analysis of OLP-related genes and metabolic information in the future.

## Data Availability Statement

The original contributions presented in the study are included in the article/**Supplementary Material**. Further inquiries can be directed to the corresponding authors.

## Ethics Statement

The studies involving human participants were reviewed and approved by Ethics Committee of the First Affiliated Hospital of Zhengzhou University. The patients/participants provided their written informed consent to participate in this study.

## Author Contributions

M-zX and Y-yS conducted the study and drafted the original manuscript. H-yZ and ZS supervised the research. M-zX, Y-yS, C-sL, and H-xM performed the experiments. M-zX, Y-yS, C-sL, and NL analyzed data. L-hZ, L-wL, Q-zD, PX, ZS, and H-yZ revised the manuscript. All authors contributed to the article and approved the submitted version.

## Funding

This study was supported by the Key Scientific Research Project of Henan Institution of Higher Education (21A320025), Henan Province Medical Science and Technology Research Plan (SBGJ202002116 and 212102310104).

## Conflict of Interest

The authors declare that the research was conducted in the absence of any commercial or financial relationships that could be construed as a potential conflict of interest.

## Publisher’s Note

All claims expressed in this article are solely those of the authors and do not necessarily represent those of their affiliated organizations, or those of the publisher, the editors and the reviewers. Any product that may be evaluated in this article, or claim that may be made by its manufacturer, is not guaranteed or endorsed by the publisher.

## References

[1] Bonar-Alvarez P Perez Sayans M Garcia-Garcia A Chamorro-Petronacci C Gandara-Vila P Luces-Gonzalez R. Correlation Between Clinical and Pathological Features of Oral Lichen Planus: A Retrospective Observational Study. Medicine (Baltimore) (2019) 98:e14614. doi: 10.1097/MD.0000000000014614 30813189 PMC6408116

[2] Gonzalez-Moles MA Warnakulasuriya S Gonzalez-Ruiz I Gonzalez-Ruiz L Ayen A Lenouvel D. Worldwide Prevalence of Oral Lichen Planus: A Systematic Review and Meta-Analysis. Oral Dis (2021) 27:813–28. doi: 10.1111/odi.13323 32144836

[3] Olson MA Rogers RS 3rd Bruce AJ. Oral lichen planus. Clin Dermatol (2016) 34:495–504. doi: 10.1016/j.clindermatol.2016.02.023 27343965

[4] Gonzalez-Moles MA Ruiz-Avila I Gonzalez-Ruiz L Ayen A Gil-Montoya JA Ramos-Garcia P. Malignant Transformation Risk of Oral Lichen Planus: A Systematic Review and Comprehensive Meta-Analysis. Oral Oncol (2019) 96:121–30. doi: 10.1016/j.oraloncology.2019.07.012 31422203

[5] Giuliani M Troiano G Cordaro M Corsalini M Gioco G Lo Muzio L. Rate of Malignant Transformation of Oral Lichen Planus: A Systematic Review. Oral Dis (2019) 25:693–709. doi: 10.1111/odi.12885 29738106

[6] Yang XY Li XZ Zhang SN. Metabolomics Analysis of Oral Mucosa Reveals Profile Perturbation in Reticular Oral Lichen Planus. Clin Chim Acta (2018) 487:28–32. doi: 10.1016/j.cca.2018.09.021 30218656

[7] Yang XY Li XZ Zhang SN. Urinary Metabolomic Signatures in Reticular Oral Lichen Planus. Heliyon (2020) 6:e04041. doi: 10.1016/j.heliyon.2020.e04041 32490246 PMC7256305

[8] Li XZ Zhang SN Yang XY. Serum-Based Metabolomics Characterization of Patients With Reticular Oral Lichen Planus. Arch Oral Biol (2019) 99:183–9. doi: 10.1016/j.archoralbio.2019.01.019 30731368

[9] Cruz AF Vitorio JG Duarte-Andrade FF Diniz MG Canuto GAB de Toledo JS. Reticular and Erosive Oral Lichen Planus Have a Distinct Metabolomic Profile: A Preliminary Study Using Gas Chromatography-Mass Spectrometry. J Oral Pathol Med (2019) 48:400–5. doi: 10.1111/jop.12842 30801783

[10] Wang XS Sun Z Liu LW Du QZ Liu ZS Yang YJ. Potential Metabolic Biomarkers for Early Detection of Oral Lichen Planus, a Precancerous Lesion. Front Pharmacol (2020) 11:603899. doi: 10.3389/fphar.2020.603899 33240093 PMC7677577

[11] Scully C Carrozzo M. Oral Mucosal Disease: Lichen Planus. Br J Oral Maxillofac Surg (2008) 46:15–21. doi: 10.1016/j.bjoms.2007.07.199 17822813

[12] Mignogna MD Fedele S Lo Russo L Lo Muzio L Bucci E. Immune Activation and Chronic Inflammation as the Cause of Malignancy in Oral Lichen Planus: Is There Any Evidence? Oral Oncol (2004) 40:120–30. doi: 10.1016/j.oraloncology.2003.08.001 14693234

[13] O'Byrne KJ Dalgleish AG. Chronic Immune Activation and Inflammation as the Cause of Malignancy. Br J Cancer (2001) 85(4):473–83. doi: 10.1054/bjoc.2001.1943 PMC2364095 11506482

[14] Clevers H. At the Crossroads of Inflammation and Cancer. Cell (2004) 118:671–4. doi: 10.1016/j.cell.2004.09.005 15369667

[15] Gutierrez S Svahn SL Johansson ME. Effects of Omega-3 Fatty Acids on Immune Cells. Int J Mol Sci (2019) 20:5028. doi: 10.3390/ijms20205028 PMC6834330 31614433

[16] Zhao Y Joshi-Barve S Barve S Chen LH. Eicosapentaenoic Acid Prevents LPS-Induced TNF-Alpha Expression by Preventing NF-kappaB Activation. J Am Coll Nutr (2004) 23:71–8. doi: 10.1080/07315724.2004.10719345 14963056

[17] Pflug KM Sitcheran R. Targeting NF-kappaB-Inducing Kinase (NIK) in Immunity, Inflammation, and Cancer. Int J Mol Sci (2020) 21:8470. doi: 10.3390/ijms21228470 PMC7696043 33187137

[18] Wang J Zhai X Guo J Li Y Yang Y Wang L. Long non-Coding RNA DQ786243 Modulates the Induction and Function of CD4(+) Treg Cells Through Foxp3-miR-146a-NF-kappaB Axis: Implications for Alleviating Oral Lichen Planus. Int Immunopharmacol (2019) 75:105761. doi: 10.1016/j.intimp.2019.105761 31325726

[19] Liu J Geng F Sun H Wang X Zhang H Yang Q. Candida Albicans Induces TLR2/MyD88/NF-kappaB Signaling and Inflammation in Oral Lichen Planus-Derived Keratinocytes. J Infect Dev Ctries (2018) 12:780–6. doi: 10.3855/jidc.8062 31999637

[20] Soleimani A Rahmani F Ferns GA Ryzhikov M Avan A Hassanian SM. Role of the NF-kappaB Signaling Pathway in the Pathogenesis of Colorectal Cancer. Gene (2020) 726:144132. doi: 10.1016/j.gene.2019.144132 31669643

[21] Tang W Wu J Jin S He L Lin Q Luo F. Glutamate and Aspartate Alleviate Testicular/Epididymal Oxidative Stress by Supporting Antioxidant Enzymes and Immune Defense Systems in Boars. Sci China Life Sci (2020) 63:116–24. doi: 10.1007/s11427-018-9492-8 31102177

[22] Banerjee S Mukherjee S Mitra S Singhal P. Comparative Evaluation of Mitochondrial Antioxidants in Oral Potentially Malignant Disorders. Kurume Med J (2020) 66:15–27. doi: 10.2739/kurumemedj.MS661009 32378537

[23] Liao H Luo Y Long L Peng J Qiu X Yuan P. Anxiety and Oral Lichen Planus. Oral Dis (2021) 27:506–14. doi: 10.1111/odi.13569 32697012

[24] Riedel WJ Sobczak S Schmitt JA. Tryptophan Modulation and Cognition. Adv Exp Med Biol (2003) 527:207–13. doi: 10.1007/978-1-4615-0135-0_24 15206734

[25] Richard DM Dawes MA Mathias CW Acheson A Hill-Kapturczak N Dougherty DM. L-Tryptophan: Basic Metabolic Functions, Behavioral Research and Therapeutic Indications. Int J Tryptophan Res (2009) 2:45–60. doi: 10.4137/ijtr.s2129 20651948 PMC2908021

[26] Pennanen L van der Hart M Yu L Tecott LH. Impact of Serotonin (5-HT)2C Receptors on Executive Control Processes. Neuropsychopharmacology (2013) 38:957–67. doi: 10.1038/npp.2012.258 PMC3629384 23303047

[27] Cowen PJ Browning M. What has Serotonin to do With Depression? World Psychiatry (2015) 14(2):158–60. doi: 10.1002/wps.20229 PMC4471964 26043325

[28] Laychock SG Tian Y Sessanna SM. Endothelial Differentiation Gene Receptors in Pancreatic Islets and INS-1 Cells. Diabetes (2003) 52:1986–93. doi: 10.2337/diabetes.52.8.1986 12882914

[29] Spiegel S Kolesnick R. Sphingosine 1-Phosphate as a Therapeutic Agent. Leukemia (2002) 16:1596–602. doi: 10.1038/sj.leu.2402611 12200669

[30] Bieberich E. Ceramide Signaling in Cancer and Stem Cells. Future Lipidol (2008) 3:273–300. doi: 10.2217/17460875.3.3.273 19050750 PMC2493052

[31] Moreno-Sanchez R Marin-Hernandez A Gallardo-Perez JC Pacheco-Velazquez SC Robledo-Cadena DX Padilla-Flores JA. Physiological Role of Glutamate Dehydrogenase in Cancer Cells. Front Oncol (2020) 10:429. doi: 10.3389/fonc.2020.00429 32328457 PMC7160333

[32] Zhang W Bouchard G Yu A Shafiq M Jamali M Shrager JB. GFPT2-Expressing Cancer-Associated Fibroblasts Mediate Metabolic Reprogramming in Human Lung Adenocarcinoma. Cancer Res (2018) 78:3445–57. doi: 10.1158/0008-5472.CAN-17-2928 PMC6030462 29760045

[33] Verbovsek U Motaln H Rotter A Atai NA Gruden K Van Noorden CJ. Expression Analysis of All Protease Genes Reveals Cathepsin K to be Overexpressed in Glioblastoma. PloS One (2014) 9:e111819. doi: 10.1371/journal.pone.0111819 25356585 PMC4214761

