# Supplementary material for: Metabolomics and Transcriptomics Analysis on Metabolic Characteristics of Oral Lichen Planus
Source: Front Oncol. 2021 Oct 19;11:769163. doi: 10.3389/fonc.2021.769163 (PMC8560742; doi:10.3389/fonc.2021.769163)
Supplement: Supplementary file 1 [file DataSheet_1.docx]

Supplementary Material


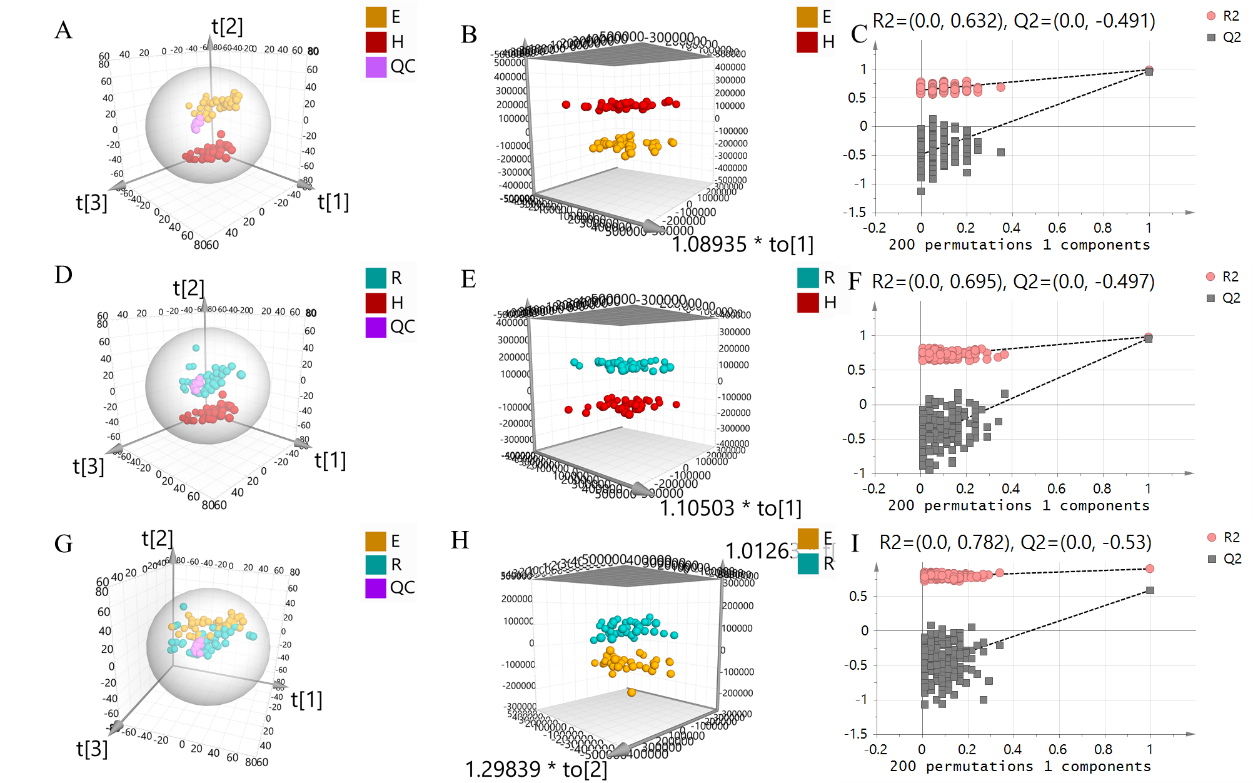


**Supplementary figure 1** Principal component analysis (PCA) diagram of serum samples of EOLP group and HC group (**A**), ROLP group and HC group (**D**), EOLP group and ROLP group (**G**) in positive ion mode, OPLS-DA score diagram (**B, E, H**) and 200 permutation test (**C, F, I**) of EOLP group and HC group, ROLP group and HC group, EOLP group and ROLP group in positive ion mode. E: EOLP; R: ROLP; H: HC.


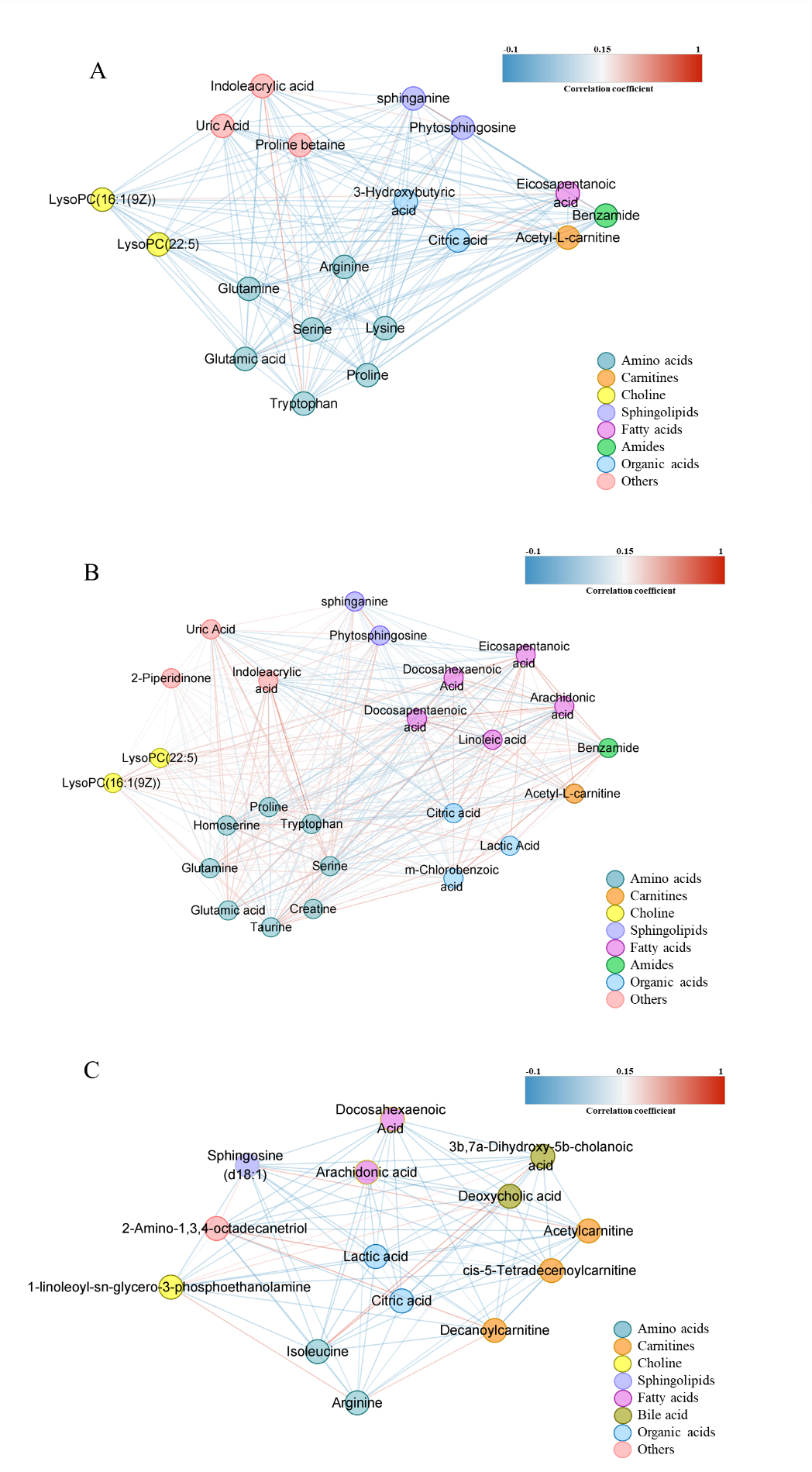


**Supplementary figure 2** EOLP/HC(**A**), ROLP/HC(**B**) and EOLP/ROLP(**C**) metabolites correlation network diagram


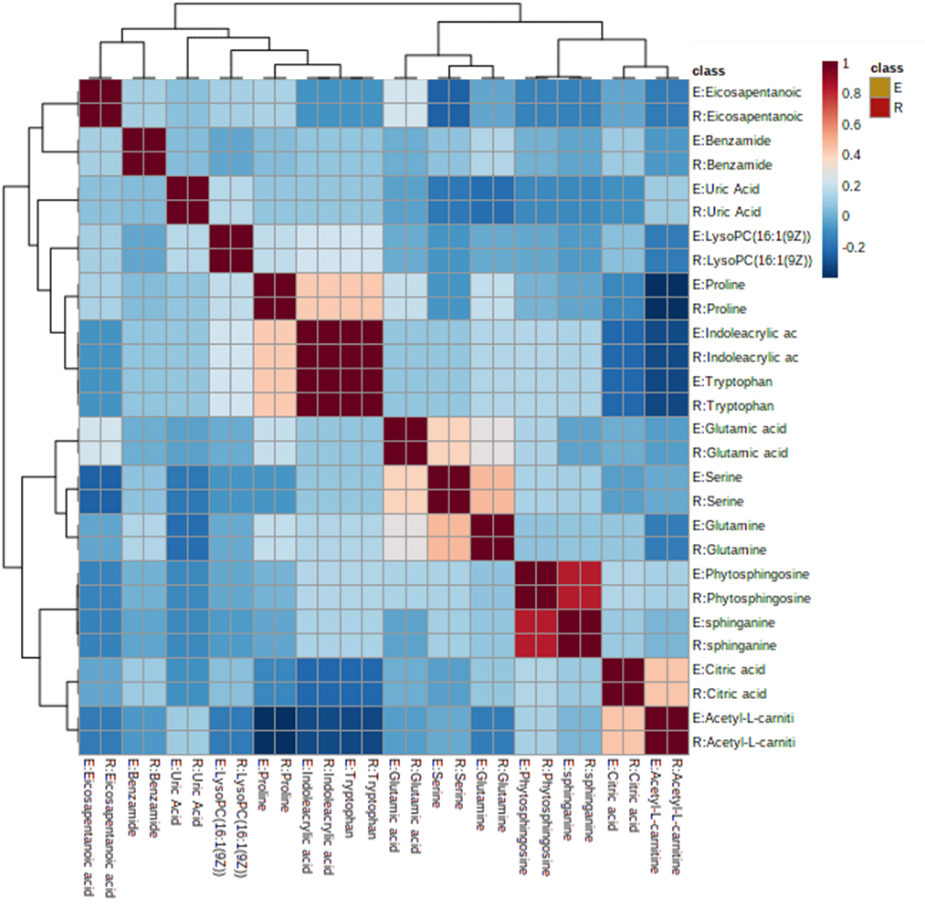


**Supplementary figure 3** Spearman correlation analysis heat map


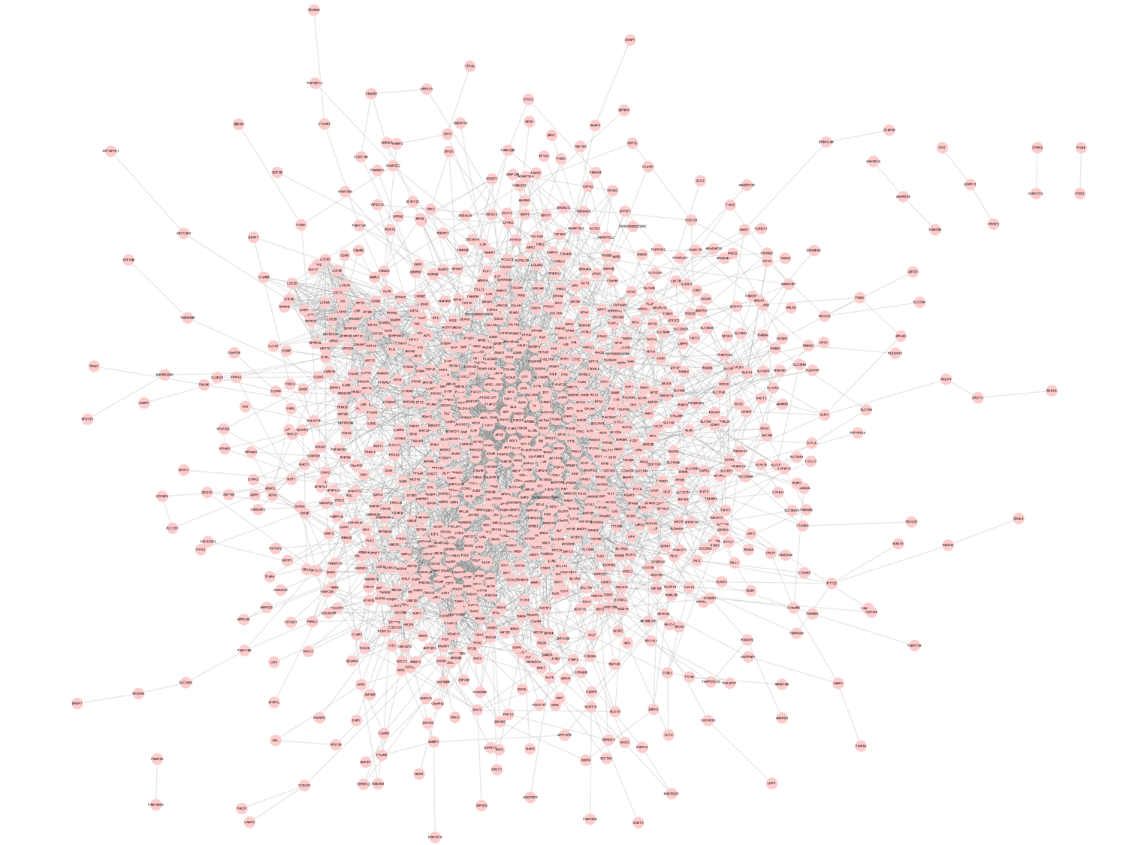


**Supplementary figure 4** GSE52130 protein interaction network diagram
